# Lower reoperation rate for cemented hemiarthroplasty than for uncemented hemiarthroplasty and internal fixation following femoral neck fracture: 12- to 19-year follow-up of patients aged 75 years or more

**DOI:** 10.3109/17453674.2013.792033

**Published:** 2013-05-31

**Authors:** Bjarke Viberg, Søren Overgaard, Jens Lauritsen, Ole Ovesen

**Affiliations:** ^1^Department of Orthopaedic Surgery and Traumatology, Odense University Hospital, Odense; ^2^Institute of Clinical Research, University of Southern Denmark; ^3^Institute of Public Health, Department of Biostatistics, University of Southern Denmark, Denmark

## Abstract

**Background and purpose:**

Elderly patients with displaced femoral neck fractures are commonly treated with a hemiarthroplasty (HA), but little is known about the long-term failure of this treatment. We compared reoperation rates for patients aged at least 75 years with displaced femoral neck fractures treated with either internal fixation (IF), cemented HA, or uncemented HA (with or without hydroxyapatite coating), after 12–19 years of follow-up.

**Methods:**

4 hospitals with clearly defined guidelines for the treatment of 75+ year-old patients with a displaced femoral neck fracture were included. Cohort 1 (1991–1993) with 180 patients had undergone IF; cohort 2 (1991–1995) with 203 patients had received an uncemented bipolar Ultima HA stem (Austin-Moore); cohort 3 (1991–1995) with 209 patients had received a cemented Charnley-Hastings HA; and cohort 4 (1991–1998) with 158 patients had received an uncemented hydroxyapatite-coated Furlong HA. Data were retrieved from patient files, from the region-based patient administrative system, and from the National Registry of Patients at the end of 2010. We performed survival analysis with adjustment for comorbidity, age, and sex.

**Results:**

Cemented HA had a reoperation rate (RR) of 5% and was used as reference in the Cox regression analysis, which showed significantly higher hazard ratios (HRs) for IF (HR = 3.8, 95% CI: 1.9–7.5; RR = 18%), uncemented HA (HR = 2.2, CI: 1.1–4.5; RR = 11%) and uncemented hydroxyapatite-coated HA (HR = 3.6, CI: 1.8–7.4; RR = 16%).

**Interpretation:**

Cemented HA has a superior long-term hip survival rate compared to IF and uncemented HA (with and without hydroxyapatite coating) in patients aged 75 years or more with displaced femoral neck fractures.

The best strategy for treatment of displaced femoral neck fractures has been discussed for years (Parker 2000, Bhandari et al. 2005, Rogmark and Johnell 2005), and the issue is becoming increasingly important in light of the growing number of elderly people with hip fractures because of increasing life expectancy (Nymark et al. 2006, Ahlborg et al. 2010, Stoen et al. 2012). Internal fixation (IF) is associated with less initial surgical trauma, less blood loss, and shorter operating time (Parker and Gurusamy 2006, Rogmark and Johnell 2006, Wang et al. 2009) but it has a high reoperation rate—varying from 10% to 57% (Heetveld et al. 2009).

In short-term studies, primary arthroplasty has been shown to have a much lower percentage of reoperations (4–32%) (Heetveld et al. 2009), and cemented prostheses have been shown to give less postoperative pain and better mobility than uncemented prostheses (Parker et al. 2010a). 2 recent meta-analyses showed the same results, but emphasized that the observations applied to older uncemented hemiarthroplasty (HA) designs (Azegami et al. 2011, Luo et al. 2012). 2 randomized controlled trials (RCTs) (Figved et al. 2009, Deangelis et al. 2012) compared a cemented HA and an uncemented hydroxyapatite-coated HA. Both RCTs showed good results for both HAs with no difference in complications, mortality, or functional outcome after 1 year.

Most RCTs that have been performed have had a maximum follow-up time of 2 years, so little is known about the long-term performance of IF and HA. 3 RCTs had a follow-up time of more than 10 years (Ravikumar and Marsh 2000, Leonardsson et al. 2010, Parker et al. 2010b) and none of them included a hydroxyapatite-coated HA. Due to increasing life expectancy, it is becoming important to know the long-term results of treatment of femoral neck fractures (von Friesendorff et al. 2008, Statistics Denmark 2012). More studies on the long-term outcome of this treatment are therefore needed.

We compared reoperation rates for 75+ year-old patients who had had displaced femoral neck fractures treated with either IF, cemented HA, or uncemented HA (with or without hydroxyapatite coating), after a follow-up time of 12–19 years.

## Patients and methods

### Patients

**Figure 1.**
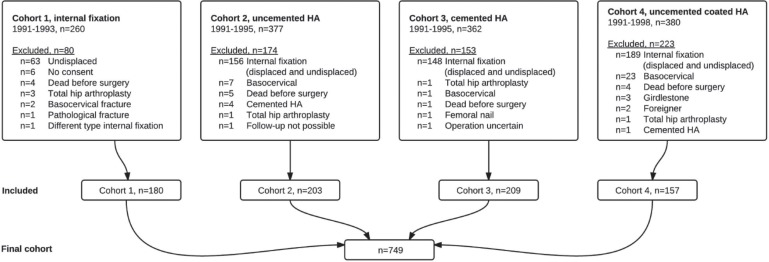
Flow chart describing the cohorts, with inclusions and exclusions. HA: hemiarthroplasty; uncemented coated: uncemented hydroxyapatite-coated.

4 hospitals with clearly defined guidelines for treatment of 75+ year-old patients with a displaced femoral neck fracture were sought. 8 hospitals using different implants were identified and 3 had the following clearly defined guidelines: IF should be used for the undisplaced fracture and HA for the displaced fracture in patients aged 75+ years. A fourth hospital that used IF for all femoral neck fractures was also included. Thus, 4 historically matching cohorts were identified at Odense University Hospital, Svendborg Hospital, Aarhus Municipal Hospital, and Hilleroed Hospital. The identity of the hospitals was hidden and the patient groups were referred to as cohorts 1–4. All patients had originally been operated or supervised by a senior registrar. The same surgical procedure (posterolateral) had been used in cohorts 2–4 (HA). In these 3 cohorts, patients with IF operations were excluded. The majority of these patients had probably had an undisplaced fracture, but since all radiographs had been destroyed, it was not possible to confirm how many fractures had been displaced (Figure 1). Postoperatively, full weight bearing exercises from day 1 had been encouraged and similar drugs for thrombosis prophylaxis and antibiotics had been given. The patients had had up to 1 year of regular follow-up after their operation.

### Cohort 1

The first cohort included patients from a previous prospective, randomized study comparing IF and a dynamic hip screw (Ovesen et al. 1997, personal communication). Exclusion criteria were pathological fracture and patient not able/willing to sign an informed consent. During the period March 1, 1991 to June 1, 1993, 260 femoral neck fracture patients had been treated at the hospital. 80 patients were excluded from the present study, mainly due to an undisplaced fracture (63), and 180 patients were included. No difference in reoperation rate was seen after 17 years of follow-up.

### Cohort 2

During the period 1991–1995, hospital 2 had used an uncemented bipolar Ultima HA, which consisted of a one-size Austin-Moore stem, 190 mm long, 135-degree neck angle, with a collar and a bipolar 42- to 56-mm Ultima head. There had been 377 femoral neck fracture patients during that time, and 156 of those were excluded due to IF operations. 203 patients were included in the present study.

### Cohort 3

During the period 1991–1995, hospital 3 had used a cemented bipolar Charnley-Hastings HA. The Charnley stem was a one-sized flanged 40, 112.4 mm long, 130-degree neck angle, and a bipolar 36- to 56-mm Hastings head was used. There had been 362 femoral neck fracture patients during that period, and IF had been used in 148 patients. 209 patients were included in the present study.

### Cohort 4

During the period 1991–1998, hospital 4 had used a bipolar uncemented hydroxyapatite-coated Furlong HA. The Furlong stem was fully coated with hydroxyapatite, with sizes of 9–16 mm, 127 degree neck angle, and had a collar. The bipolar head came in sizes of 40–58 mm. There had been 380 femoral neck fracture patients in that period and IF had been performed in 189 patients. 223 patients were excluded and 157 patients were included.

Thus, 749 patients from the 4 hospitals were included in the present study (Figure 1). The number of patients at risk was 471 after 2 years, 375 after 5 years, and 199 after 10 years (Table 1).

**Table 1.** Reoperations by implant. For patients at risk, attrition was mostly due to high mortality. Values are number of reoperations/patients at risk

	Year	Year	Years	Years	Years
	1	2	3–5	6–10	11–19
Internal fixation	25/180	4/105	3/82	1/41	0/13
Uncemented HA	14/203	4/125	3/99	1/54	0/15
Cemented HA	7/209	0/147	2/120	2/65	0/19
Uncemented coated HA**^ a^**	12/157	2/94	7/74	2/39	2/9
Total	58/749	10/471	15/375	6/199	2/56

HA: hemiarthroplasty. **^a ^**Hydroxyapatite coated.

### Data

Patients were identified through procedure books and the region-based patient administrative system. Information on operation (date, side, type), reoperation (date, side, type), and date of death was recorded. In Denmark, all residents have a unique personal identity number from the Civil Registration System, which contains data on vital status and residence for the entire Danish population (Frank 2000). The identity number enabled us to retrieve data on all patients from the National Registry of Patients (NRP), which was done on November 9, 2010. The NRP was established in 1977 and contains data on all admissions and discharges from hospitals in Denmark, including dates of admission and discharge, surgical procedures performed, and up to 20 diagnoses for every discharge. The coding from the NRP has a consistently high positive predictive value (Thygesen et al. 2011) and was used to create a Charlson comorbidity index (Charlson et al. 1987) with diagnosis codes up to 10 years preceding the date of operation of a patient. The NRP also contained information about the reoperation data, and all reoperations were confirmed in the patient files.

Failure was defined as any procedure that led to a major reoperation with loss/change of hip implant or periprosthetic/new fracture. A new fracture was defined as subtrochanteric at the level of IF implant or a femoral neck fracture more than 1 year after removal of IF. Reasons for failure were recorded as stated in the patient files or according to codes in the NRP. Patients were followed until first reoperation or until death, whichever came first. Minor procedures were defined as closed or open reduction (including change of bipolar head) and removal of IF. The codes for minor procedures were also extracted from the NRP, but as not all patients were admitted or coded correctly in that time period, there was some uncertainty about the completeness and accuracy of these codes, and therefore data on minor procedures were not included in this study.

### Statistics

The statistical software program STATA 11 was used for the analysis. The term rate is used as proportion rather than outcome per time unit. A chi-square test for the categorical variables was used for group comparison before survival analysis. Data were set as survival data, and group comparisons with log-rank tests and Kaplan-Meier graphs were performed. The proportional-hazards assumption was evaluated statistically (goodness of fit) and graphically using log-log Kaplan-Meier survival plot against survival time. Cox regression analysis was used with adjustment for comorbidity (Charlson index), sex, and age. The Charlson comorbidity index score was categorized as done in the Danish Registry of Hip Fractures (Dansk Tværfagligt Register for Hoftenære Lårbensbrud 2011) (0, 1, 2, and 3 or more points) and age was also categorized in 5-year intervals (75, 80, 85, and 90 or more). To ascertain a possible theoretical influence of non-independence in patients with bilateral femoral neck fractures, a sensitivity test was performed on the Cox regression analysis excluding the data on the second femoral neck fracture.

## Results

The cohorts were similar with regard to age, sex, comorbidity, and survival (Table 2). Patients treated with a cemented HA (cohort 3) had the lowest overall reoperation rate, of 5%, followed by uncemented HA and uncemented hydroxyapatite-coated HA (Table 2). For IF, the number of reoperations was 33 (Table 2) and most of these had been performed within the first 2 years after the primary operation (Table 1), leaving 82% of the patients with their natural hip.

**Table 2.** Key patient demographics for 749 patients in the 4 cohorts

	Cohort 1	Cohort 2	Cohort 3	Cohort 4	
	Internal	Uncemented	Cemented	Uncemented	
	fixation	HA	HA	coated HA	p-value
No. of patients	180	203	209	157	–
Median age (IQR)	83 (79–87)	84 (80–87)	83 (79–88)	85 (80–89)	0.2
Sex, females/males	129/51	163/40	169/40	127/30	0.09
Median CCI score (IQR)	2 (0–3)	1 (0–3)	1 (0–3)	1 (0–3)	0.4
Median patient survival, years (IQR)	2.8 (0.1–9.9)	2.5 (0.1–9.7)	2.9 (0.1–10.7)	2.2 (0.0–9.0)	0.5
Failure (%)	33 (18.3)	22 (10.8 )	11 (5.3)	25 (15.9)	See Table 5

HA: hemiarthroplasty; IQR: interquartile range; CCI: Charlson comorbidity index.

**Figure 2.**
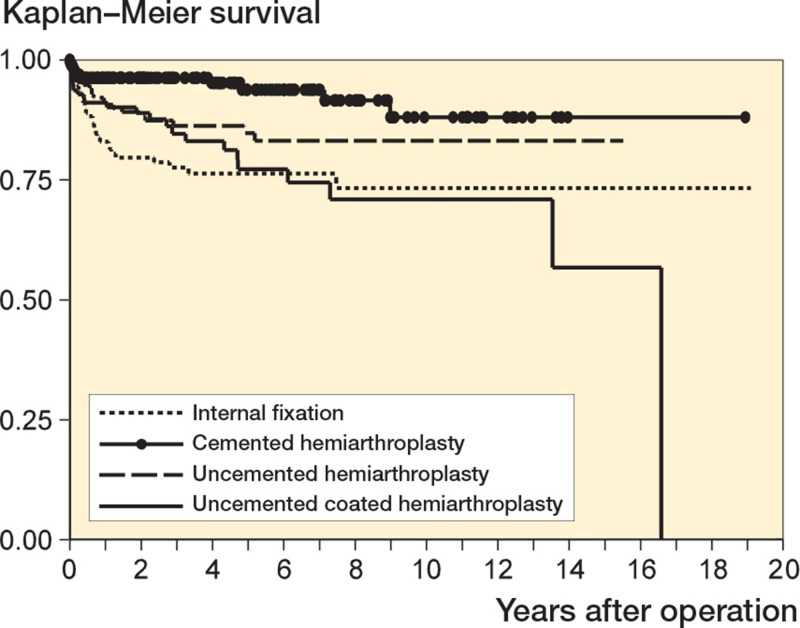
Kaplan-Meier implant survival curves, by type of operation.

The cohorts had statistically significantly different reoperation rates (log-rank test) (Figure 2). A chi-square analysis comparing the reoperation rates before and after 2 years (Table 1) showed no significant difference for IF and uncemented HA (p < 0.2), but there were proportionally higher reoperation rates after 2 years for cemented HA (p < 0.001) and uncemented hydroxyapatite-coated HA (p < 0.001).

For IF, 28 of the 33 failures had been osteosynthesis failure (Table 3). Periprosthetic fractures had been the main reason for reoperations of HA with similar rates (13/22, 6/11, and 14/25 of the reoperations) (Table 4).

**Table 3.** Reasons for reoperation

	Internal	Uncemented	Cemented	Uncemented
	fixation	HA	HA	coated HA
Osteosynthesis failure	28	0	0	0
Arthrosis	4	2	0	0
Dislocation	0	3	5	8
Loosening	0	2	0	0
Periprosthetic fracture	0	13	6	14
Infection	1	2	0	2
Unknown	0	0	0	1
Total	33	22	11	25

HA: hemiarthroplasty.

**Table 4.** Type of reoperation

	Internal	Uncemented	Cemented	Uncemented
	fixation	HA	HA	coated HA
Total hip arthroplasty	24	8	5	13
Cemented HA	6	3	0	3
Girdlestone	2	1	0	1
Osteosynthesis	0	10	6	8
Re-osteosynthesis	1	0	0	0
Total	33	22	11	25

HA: hemiarthroplasty.

The Cox regression analysis using IF as reference revealed a significantly lower hazard ratio (HR) for cemented HA but not for uncemented HA or uncemented hydroxyapatite-coated HA (Table 5). In the Cox regression analysis that followed, cemented HA was used as reference in order to evaluate whether cemented HA had a different HR from the other HAs. The analysis showed significantly higher HRs for IF, uncemented HA, and uncemented hydroxyapatite-coated HA than for cemented HA. The analyses were adjusted for comorbidity, age, and sex (all non-significant). A sensitivity test excluding the patient’s second fracture (n = 25) showed only minor changes in HRs, confidence intervals, and p-values.

**Table 5.** Survival analysis of hip failure adjusted for sex, comorbidity, and age (all non-significant)

	HR	95% CI	p-value	HR	95% CI	p-value
Internal fixation	1 (ref)			3.8	1.9–7.5	< 0.001
Uncemented HA	0.6	0.3–1.0	0.05	2.2	1.1–4.5	0.04
Cemented HA	0.3	0.1–0.5	< 0.001	1 (ref)		
Uncemented coated HA	1.0	0.6–1.6	0.9	3.6	1.8–7.4	< 0.001

HR: hazard ratio; CI: confidence interval; HA: hemiarthroplasty.

## Discussion

We found a lower reoperation rate (18%) after IF at 19 years than has been found in meta-analyses, which found reoperation rates of 36% (Parker and Gurusamy 2006, Rogmark and Johnell 2006, Wang et al. 2009), and compared to other long-term outcome studies of IF (Ravikumar and Marsh 2000, Leonardsson et al. 2010, Parker et al. 2010b), which have found reoperation rates of 33–46%. Our finding might be explained by the fact that hospital 1 was a large teaching hospital with approximately 500 hip fractures a year and that it had used IF almost exclusively for all femoral neck fractures for at least a decade before the study period. All surgical procedures were also done or supervised by specialists. Furthermore, Denmark as a nation has low reoperation rates after displaced femoral neck fracture, and in the latest report from the National Hip Registry the reoperation rate was 18% (Dansk Tværfagligt Register for Hoftenære Lårbensbrud 2011). Minor procedures such as closed or open reduction (including change of bipolar head) and removal of IF were not included in the present study, and they must be taken into account when comparing IF and HA results.

During the last 3 decades, a variety of different types and concepts of HA have been used. In the present study, 3 different concepts were used: a bipolar uncemented HA (Ultima/Austin-Moore), a bipolar cemented HA (Charnley-Hastings), and a bipolar uncemented hydroxyapatite-coated HA (Furlong). The reoperation rates for cemented and uncemented HA have been comparable in RCT studies (Parker et al. 2010a, Azegami et al. 2011, Luo et al. 2012), even though the uncemented Austin-Moore stem has had inferior outcome in other types of studies (Rogmark et al. 2012). No large differences between the groups in the present study were apparent until 3–4 years had elapsed (Figure 2). An RCT with 13 years of follow-up (Ravikumar and Marsh 2000) found a reoperation rate for uncemented HA of 24%, as compared to 11% in the present study. However, one RCT using an uncemented HA with a follow-up of 9–15 years found a reoperation rate of only 7% (Parker et al. 2010a). The difference in the reoperation rates between the study by Parker et al. (2010a) and our study could be a result of the nationwide search for reoperations through the NRP that was done in our study. The older uncemented HAs are still widely used globally whereas the Ultima/Austin-Moore HA has almost been phased out in the Scandinavian countries (Nasjonalt Hoftebruddregister 2011, Leonardsson et al. 2012).

One RCT comparing a cemented HA with an uncemented hydroxyapatite-coated HA found similar reoperation rates (Figved et al. 2009). The study showed a reoperation rate after 1 year of 7% in the uncemented group (6% in the cemented group), which is comparable to our findings after 1 year (12/157 = 8%). However, the present study showed that half of the reoperations occured after 1 year, and the final rate was 16%. The high reoperation rate in this study could be due to the Furlong stem. In comparison, the study by Chandran et al. (2006) found a reoperation rate of only 8% in 112 patients after a follow-up of 3–14 years. Livesley et al. (1993) compared an uncemented HA (Austin-Moore) with an uncemented hydroxyapatite-coated HA (Furlong) and found no significant difference in outcome after 1 year. A newly published study from the Norwegian Hip Fracture Register (Gjertsen et al. 2012) showed a 5-year survival of 97% for cemented HA, which was statistically significantly different from the 91% survival for all the uncemented HAs (which were almost exclusively hydroxyapatite-coated HA). This tendency is confirmed in the present study.

The present study had some limitations. Firstly, there were some deviations from the guidelines for cohorts 2–4, as a small proportion of the displaced fractures had been treated with IF, thus introducing a small selection bias. Secondly, 2 different IF implants had been used in cohort 1, but this is not likely to have affected our results; Bhandari et al. (2009) showed no difference in reoperation rate between the 2 implants. Thirdly, due to the low number of patients at risk after 10 years of follow-up, the results hereafter can only be considered to be indicative.

The study also had several strengths. Firstly, there was a long follow-up time. In spite of the fact that many patients with femoral neck fractures have comorbidities, the life expectancy of an average 75-year-old woman is 7 years both in Denmark (Statistics Denmark 2012) and in the UK (Office for National Statistics 2011), which suggests that life expectancy may also be longer for patients with a fracture (von Friesendorff et al. 2008). Secondly, all reoperations were validated at the case level using 4 matching cohorts with comparable guidelines but different implant types. Thirdly, few patients were lost to follow-up and all reoperations were found using a link to the NRP, which also made it possible to adjust for comorbidity. Lastly, all HAs were bipolar and there were therefore no potential confounders from the unipolar HA.

In conclusion, reoperation rate and hazard ratio were lower for cemented HA than for IF, uncemented HA, and uncemented hydroxyapatite-coated HA in 75+ year-old femoral neck fracture patients after up to 19 years of follow-up. Our findings therefore suggest that cemented HA is the best treatment for a displaced femoral neck fracture in this patient group.
